# Psychosocial factors and their predictive value in chiropractic patients with low back pain: a prospective inception cohort study

**DOI:** 10.1186/1746-1340-15-5

**Published:** 2007-03-29

**Authors:** Jennifer M Langworthy, Alan C Breen

**Affiliations:** 1 Institute for Musculoskeletal Research and Clinical Implementation, Anglo-European College of Chiropractic, 13-15 Parkwood Road, Bournemouth, BH5 2DF, UK

## Abstract

**Background:**

Being able to estimate the likelihood of poor recovery from episodes of back pain is important for care. Studies of psychosocial factors in inception cohorts in general practice and occupational populations have begun to make inroads to these problems. However, no studies have yet investigated this in chiropractic patients.

**Methods:**

A prospective inception cohort study of patients presenting to a UK chiropractic practice for new episodes of non-specific low back pain (LBP) was conducted. Baseline questionnaires asked about age, gender, occupation, work status, duration of current episode, chronicity, aggravating features and bothersomeness using Deyo's 'Core Set'. Psychological factors (fear-avoidance beliefs, inevitability, anxiety/distress and coping, and co-morbidity were also assessed at baseline. Satisfaction with care, number of attendances and pain impact were determined at 6 weeks. Predictors of poor outcome were sought by the calculation of relative risk ratios.

**Results:**

Most patients presented within 4 weeks of onset. Of 158 eligible and willing patients, 130 completed both baseline and 6-week follow-up questionnaires. Greatest improvements at 6 weeks were in interference with normal work (ES 1.12) and LBP bothersomeness (ES 1.37). Although most patients began with moderate-high back pain bothersomeness scores, few had high psychometric ones. Co-morbidity was a risk for high-moderate interference with normal work at 6 weeks (RR 2.37; 95% C.I. 1.15–4.74). An episode duration of >4 weeks was associated with moderate to high bothersomeness at 6 weeks (RR 2.07; 95% C.I. 1.19 – 3.38) and negative outlook (inevitability) with moderate to high interference with normal work (RR 2.56; 95% C.I. 1.08 – 5.08).

**Conclusion:**

Patients attending a private UK chiropractic clinic for new episodes of non-specific LBP exhibited few psychosocial predictors of poor outcome, unlike other patient populations that have been studied. Despite considerable bothersomeness at baseline, scores were low at follow-up. In this independent health sector back pain population, general health and duration of episode before consulting appeared more important to outcome than psychosocial factors.

## Background

Recovery from persistent low back pain is determined not solely by clinical factors but also by the individual's psychological state [1]. Such psychological and social factors have come to be considered important in general practice and occupational back pain populations [2]. However, chiropractic investigators have given these less attention. This is not to suggest that chiropractors themselves regard these issues as unimportant. In a recent survey of 1,045 chiropractors [3], 80–90% reported their belief that emotional factors influence pain syndromes. However, less than half said they were able to evaluate these factors while just one-third felt able to treat them.

Failure to improve as expected leads to disappointment and sometimes to unexpectedly protracted treatment. As well as the distress this may cause the patient, it could engender criticism of the practitioners and their profession. Indeed, the chiropractic profession has been noted for seeming over-treatment of patients [4]. Traditional non-physical treatment approaches used by chiropractors include counselling, ergonomic and other advice [5], plus the alleviation of stress [6]. Chiropractic researchers, however, have tended to use mainly severity measures to predict poor outcomes [7] and calls for deeper understanding of these issues have been made from within this research community [8-10]. If chiropractors had access to information about the role of psychosocial risk factors in their patients, they may be able to develop better targeted and justified treatment strategies.

### The nature of pain

Pain is defined as an unpleasant sensory and emotional experience [11]. It is highly subjective being dependent on the individual's personal perceptions and therefore cannot be standardised as people respond in different ways to similar physical pain. The presence of adverse psychological factors, such as anxiety, fear or distress/depression, may have the effect of intensifying the perceived severity of pain and may play an important role in progress towards chronic pain and disability [12].

Acute pain gives rise to anxiety about its aetiology and prognosis [11], whereas chronic pain is distressing and may reinforce fears that the cause is serious and untreatable. In some individuals this may lead to feelings of helplessness and hopelessness and to withdrawal from social interaction. Beliefs also influence our perceptions of events, affect the way we cope and therefore impact significantly on an individual's response to pain and treatment [13].

### Research in other populations

Better care strategies for patients at risk of poor outcome require that they first be recognised as being at risk. The literature suggests that the main psychosocial risk factors relate predominantly to depression, distress and role issues, especially with regard to work [1,14-17]. Waddell, Burton and Main [18] found that the strongest psychosocial and socio-demographic predictors of chronic pain and disability were older age, poor general health/perceptions, abnormal pain behaviour, unemployment and expectations about return to work. However, different stakeholders may prioritise different outcomes. For example, pain relief may be the patient's priority, while return-to-work may be that of the employer and cost-of-care that of the insurer.

Research has identified risk factors with predictive value for chronicity in various public sector settings [19], notably in general practice [20] and physical therapy populations [21]. There is much less evidence in relation to chiropractic patients. Work-based strategies are effective, especially for sub-acute back pain [22] but are expensive and generally unavailable to small companies and the self-employed. Thus many such patients seek the help of chiropractors. These, however, may be different to patients in the public sector and we have no systematic knowledge of which patients who consult chiropractors are at risk of poor outcome in this population. The current study sought to discover which of the currently considered biopsychosocial risk factors for chronic disability are prominent and predict poorer outcome in non-specific back patients seeking help from chiropractors in the independent health sector.

## Methods

### Recruitment

Over a one-year period, 200 consecutive new patients contacting a chiropractic clinic in the market town of Salisbury in the UK for an appointment were asked by the clinic receptionists to confirm if their primary complaint was a new episode of pain in the lower back. If it was, they were informed about the study and that, if eligible and willing, their participation would involve the completion of two questionnaires prior to treatment on first attendance at the clinic, followed by one further, short (6 questions) questionnaire six weeks after initial presentation. This follow-up questionnaire was administered via the telephone. Two years on from initial presentation, all patients who had completed the 6-week follow-up were contacted to see if they would be willing to complete the 6-item questionnaire one more time.

### Eligibility and consent

If the patient was willing in principle to participate, the receptionist was required to ask four questions to determine eligibility. To be eligible, patients had to be aged between 18 and 65 years, to have not undergone previous back surgery, not be pregnant and to not have pain below the knee.

If the patient remained eligible and willing to join the study, s/he was asked to attend the clinic 20 minutes prior to their scheduled appointment with the chiropractor. Upon arrival, the patient was given a consent form to read and sign. The receptionist was only allowed to answer clarifying questions if asked. The patient was also given an information sheet that they could take away with them detailing the study. A contact number for the senior researcher was provided on this form in case the patient had any queries or concerns. On completion of the questionnaires, the patient was then examined by the chiropractor who confirmed their final eligibility as having non-specific back pain. Reasons for non-eligibility were recorded.

When the receptionist received back the patient notes and eligibility form, the details of those patients for whom it was appropriate to join the study were entered onto a patient log. This recorded patient contact details, date of recruitment, the granting of consent and the completion date of the first questionnaires. Completed questionnaires, signed consent and eligibility forms and the patient logs were retrieved from the clinic on a weekly basis. A date for a 4 and 6-week follow-up for each participating patient was calculated. The purpose of the 4-week follow-up was to check that the original diagnosis of non-specific (simple) back pain had not changed. This was done by review of the patient's clinical notes at the practice. The results of this re-examination of the patient's notes were also recorded in the log.

### Data collection

The baseline questionnaire requested information about age, gender, occupation, work status (part-time or full-time, shift work or not, whether the patient enjoyed their work and how much time, if any, they had taken off work with back pain in the past 3 years). Duration of current episode and chronicity [23] were determined, along with a standardised 'Core Set' of outcome measures that included bothersomeness, interference with work, attitude to persistent pain, days of reduced activities and days off work or school in the past 4 weeks [24]. The presence of aggravating features was also investigated. In addition, patients were asked to complete the Fear-Avoidance Beliefs Questionnaire (FABQ) [25], the inevitability scale of the Back Beliefs Questionnaire (BBQ) [26], the anxiety and coping scales of the Coping Strategies Questionnaire (CSQ) [27] and the 12-item version of the General Health Questionnaire (GHQ-12) [28] as a measure of psychological distress. At 6 week follow-up, pain impact was measured as at baseline, along with satisfaction with care on a 5-point numerical scale.

### 6 week follow-up

When the date for the 6 week follow-up was known, the patient was sent a reminder card showing the date and time for its completion. The time of day was determined by the patient's stated preference on the consent form as being the most convenient time for them to be contacted by telephone. This was also the case regarding where they were to be contacted, i.e. at home, work or on their mobile telephone. The patient was asked to keep the reminder somewhere prominent and to contact the investigators if the scheduled appointment was not convenient. An alternative appointment could be made for a period of up to 5 days from the original due date. On the agreed date and time, the patient was contacted by telephone and the questionnaire administered. The due date for completion of the 6 week follow-up was noted on the patient log, as was the date it was actually completed.

At 2 years, the number of attendances after the first 6-weeks from presentation was recorded from a note search. In addition, patients were contacted by telephone and asked to complete Deyo's core set [24] again, but without the item about satisfaction with care.

### Analysis

Descriptive analysis of the baseline characteristics of participating patients was initially performed, followed by correlation analysis between selected baseline and follow-up variables. Baseline and 6 week outcomes (bothersomeness, cut down days for activity and for work and satisfaction with care) were compared and effect sizes [29] were calculated. Independent 2-sample t-tests were used to compare interval data from population subgroups. The follow-up outcomes of bothersomeness and interference with work were then dichotomised, with all scores of moderate and above taken as higher severity. Scores relating to psychosocial variables were averaged and then dichotomised, with a cut-off from 50% and above. Risk ratios were then calculated between baseline and follow-up variables, including the effect of co-morbidity.

## Results

Of the 200 patients initially approached to participate in the study, 158 were eligible to participate and completed the baseline questionnaires. (Ineligibility was mainly by reason of having pain below the knee.) Of these, 130 (82%) completed the 6 week follow-up. However, 29 patients failed to provide full details at this stage, thus reducing the number of participants to 101 (64%). At 2 years, only 55 (54%) of these 101 subjects were available for further follow-up.

Fifty-seven percent of the sample was female and the mean age was 43 years (SD:10.39). The majority (n = 34) had experienced their current episode of low back pain for 1–4 weeks and 26 for >12 weeks. The remainder reported durations of 1–6 days (n = 24) and 5–12 weeks (n = 17). The current episode was the first ever experience of LBP in 24% of subjects, while over half (55%) had been troubled by episode(s) of LBP for ≥ 50% of the past year. Eighty-six percent were either employed or self-employed and virtually all (99%) enjoyed their work. Nearly two-thirds (n = 10) of the 16 patients who reported currently being off work had been off for more than 1 week and 38% of the participants also had other conditions.

Baseline outcome scores are summarised in Table 1. Sixty-three percent described their low back pain as having been moderately to extremely bothersome over the past week. Thirty-seven percent of the sample had moderate-extreme leg pain (above the knee). Moderate-extreme interference with normal work was reported by 73% and 92% were dissatisfied with their current state of well-being. The mean number of days of restricted activities and absence from work or school was, however, low in light of these levels of bothersomeness.

**Table 1:** Effect size between baseline and 6 week outcome scores

(n = 101)			
**ITEM**	**BASELINE MEAN (SD)**	**6 WEEKS MEAN (SD)**	**EFFECT SIZE 6 WEEKS**

LBP bothersomeness in past week *(max 5.0)*	3.81 (1.07)	1.99 (0.93)	1.37
Leg pain bothersomeness in past week *(max 5.0)*	2.24 (1.37)	1.42 (0.75)	0.63
Interference with normal work *(max 4.0)*	2.23 (1.10)	0.77 (0.96)	1.12
Satisfaction with current status *(max 4.0)*	0.65 (0.71)	2.08 (1.40)	0.99
Days of cut-down activity in past 4 weeks *(max 28 days)*	5.61 (7.66)	4.25 (7.66)	0.17
Days of absence from work in past 4 wks due to LBP *(max 28 days)*	3.23 (7.31)	1.43 (5.35)	0.20

The range and mean baseline psychometric scores are shown in Table 2. These generally tended toward the low end of all scales. At six weeks, 23% reported moderate-extreme LBP bothersomeness during the past week and Table 1 shows the effect sizes (ES) for the outcome variables between baseline and 6 weeks. Cut-down days in activity and work improved the least, although actual reduction in and interference with normal work were high (ES 1.12). At baseline, for at least 1 day over the previous four weeks, 62% and 26% of respondents respectively had had to cut down on their usual activities (mean 5.61 days) or had been prevented from going to work (mean 3.23 days). Largest effects were in the reduction of LBP bothersomeness (ES 1.37 at 6-weeks), whilst satisfaction with current status also had a substantial effect (ES 0.99). At six weeks, subjects rated their satisfaction with overall care at a mean of 3.55 (range 0 very dissatisfied – 4 very satisfied). Co-morbidity was found to be significantly associated with high interference with work at 6 weeks (RR = 2.37; 95% C.I. = 1.15 to 4.74), as was an episode of LBP of >4 weeks duration and a high level of interference with normal work reported at baseline (Table 3). The mean number of care encounters after the first 6 weeks from initial presentation was 1.31 for patients without co-morbidity (n = 29) and 4.49 with co-morbidity (n = 14) (p = 0.014: 2-way unpaired t-test). None of the aggravating features of LBP were significantly associated with poor outcome 6 weeks from presentation. Subjects exhibiting a negative outlook at baseline regarding the future impact of their LBP were 2.5 times more likely to experience interference with their normal work at 6 weeks (RR = 2.56; 95% C.I. = 1.08 – 5.08).

**Table 2:** Psychometric scores at baseline

**Scale**	**Range**	**Mean (SD)**
FABQ Activity	0–24	13 (5.65)
FABQ Work	0–42	13 (10.22)
FABQ Total Score	0–66	26 (12.92)
BBQ (Inevitability)	9–45	32 (6.73)
CSQ (Anxiety)	0–36	5 (6.26)
GHQ-12	0–36	13 (5.47)

**Table 3:** Relative risk of poor outcome 6 weeks from presentation

	**Bothersomeness**		**Interference with Work**	
**Baseline Variable**	**Relative Risk**	**95% C.I.**	**Relative Risk**	**95% C.I.**

Age >55 years	0.63	0.11 – 3.01	0.37	0.06 – 1.67
Duration >4 weeks	2.07*	1.19 – 3.38	1.78	0.84 – 3.73
Chronicity (LBP present >50% of past year)	1.22	0.49 – 2.71	0.92	0.34 – 2.19
High bothersomeness	0.76	0.36 – 1.66	1.96	0.45 – 11.27
High interference with normal work	1.71	0.69 – 4.57	3.42*	1.00 – 12.86
Co-morbidity	1.89	0.89 – 3.61	2.37*	1.15 – 4.74
LBP aggravated by:				
- Sitting	0.76	0.36 – 1.67	1.53	0.66 – 3.80
- Standing	0.28	0.14 – 0.47	0.71	0.32 – 1.53
- Walking	1.42	0.63 – 3.00	0.55	0.23 – 1.22
- Bending	0.43	0.21 – 0.98	1.05	0.42 – 3.19
- Lifting	1.15	0.31 – 4.68	-**	-
- Lying	1.89	0.89 – 3.67	0.86	0.42 – 1.95
Fear-avoidance (FABQ)	-***	-	-***	-
Inevitability (BBQ)	2.27	0.97 – 4.42	2.56*	1.08 – 5.08
Anxiety (CSQ)	-****	-	-****	-
General Health (GH-12)	1.02	0.20 – 3.48	1.21	0.22 – 3.88

* Statistically significant risk.

** No cases reporting moderate to high interference with work were aggravated by lifting.

*** Only one case exhibited high fear-avoidance at baseline. Despite this, LBP was reported as not bothersome at 6 weeks.

**** No cases exhibited anxiety at baseline.

At 2 years, the high attrition rate rendered further analysis to identify predictors of poor outcome unfeasible. However, 15% (n = 8) reported moderate-extreme LBP bothersomeness, while 9% (n = 5) were experiencing similar levels of interference with their normal work due to LBP. Sixteen percent (n = 9) had reduced normal activities over the preceding 4 weeks (mean = 1 day) and 4% (n = 2) had needed to take time off work due to their LBP (mean = 0.5 days).

## Discussion

### Patients

One factor which may partly explain our inability to identify any psychosocial predictors of poor outcome in LBP patients may be the relatively low number of participants. It is also possible that this inability relates to the practice being in the independent sector. Studies [30] from the public healthcare sector report high scores on psychosocial assessment and distress to be significantly associated with non-recovery at one-year. However, in the current study, participants were recruited from private practice and were not particularly distressed at the time of initial presentation. Nor were the majority overly work-disabled by their condition despite reported high bothersomeness scores. Yet these are two common predictors of poor outcome in other studies [19]. Although a proportion of patients reported moderate to extreme interference with normal work due to LBP at baseline, this was not severe enough to stop the majority from working and certainly not for any protracted length of time. Virtually all who were off work returned to work, which may be due to promptness in seeking care. One large study of chiropractic practice in Europe [5] found that the majority of patients sought care within the first 4 weeks of onset and a further study that apparent high levels of satisfaction bear little relation to the degree of functional improvement achieved [31].

### Outcome measures

The Deyo 'Core Set' of outcome measures proposed for low back pain research [24] was used in this study. This is a short, 6-item questionnaire which we chose for its conciseness within an otherwise large questionnaire battery [32]. The main measure of pain symptoms in this questionnaire was how much the pain bothered the patient. In a study seeking to classify primary care patients from general practice with low back pain, Dunn & Croft (2005) [33] found bothersomeness to be a valid measure of severity, being associated with measures of pain, disability, psychological health and work absence. Following its use as a measure of pain severity in a large UK back pain trial [34], Parsons et al (2006) [35] also used bothersomeness (anglicising the term to troublesomeness) in their comparative study which evaluated the troublesomeness of chronic, multi-site pain within individuals. In their validation study of the 'core set', Ferrer at al [32] concluded that it had the potential to be a useful tool in conjunction with other well-established outcome measures in future studies of LBP. However, they also concluded that, as their subjects and their back conditions were not typical of those presenting most commonly in primary care, further validation was needed before it could be widely recommended across LBP populations. While the 'core set' has been proposed as a concise research instrument, both Ferrer et al [32] and Dunn & Croft [33] concluded that further work is needed to verify the usefulness of bothersomeness in clinical practice. Moreover, if it is ultimately to be widely used as a single measure of severity, it will be important to be confident that both patients and clinicians interpret the term in the same way.

### Predictors of outcome

Dunn and Croft (2005) [33] found that higher back pain bothersomeness at baseline in a UK general practice population predicted a greater risk of work absence at 6 months. Our chiropractic population did not, however, have appreciable work absence despite the level of bothersomeness. Moreover, although reported high levels of interference with normal work due to LBP at baseline was significantly associated with high levels of interference with work at 6 weeks the 95% confidence interval was very wide, suggesting that this finding should be interpreted with some caution.

The proportion in our sample with work absence, however, may be untypical. Sorensen et al (2006) [36], conducted a large survey of Danish chiropractic patients, (most of whom also consulted early and for low back pain) and found that that nearly a third had been off work, compared to our 16%. Most of their work loss was, as with our sample, of less than a week's duration. Our results also suggest that early intervention may be an important factor in successful care. While distress and depression are generally considered to be among the major predictors of poor outcome [2], these are, in any case, particularly prevalent when back pain has become chronic [37]. Although little is known about predictors of outcome that may be apparent in the very early stages, one small, but carefully controlled inception cohort study in general practice in France [38] found that delayed recovery (by 3 months) was associated with higher baseline disability and low self-related health. In the current study, the first of these was not a predictor. However, co-morbidity was associated with higher levels of interference with normal work at 6 weeks and it has been suggested that greater attention should be given to the existence of co-morbidities in the treatment of non-specific LBP [39].

## Conclusion

Despite relatively high baseline bothersomeness scores, almost all patients in this study had resolution or near-resolution 6 weeks from presentation. Only co-morbidity and complaint duration of >4 weeks prior to consultation significantly predicted low back pain bothersomeness at 6 weeks. Although in this study higher inevitability scores were a significant psychological risk factor, no others were found. Studies that seek psychosocial predictors of poor long-term outcome in private chiropractic patients from measures used in this study may not find them. Future work could helpfully address whether locus of control and self-efficacy differs between patients in the public and independent healthcare sectors and whether there is a relationship between this and outcomes.

## Competing interests

The author(s) declare that they have no competing interests.

## Authors' contributions

Both authors collaborated on the rationale and design of the study and liaised with the practice for the collection of data. AB carried out the note searches for co-morbidity and numbers of treatment sessions. JL carried out all data entry. Both authors participated in the analysis of data. JL wrote the first draft of the manuscript. Both authors read and approved the final manuscript.
